# Supplementary material for: Frenetic, under-Challenged, and Worn-out Burnout Subtypes among Brazilian Primary Care Personnel: Validation of the Brazilian “Burnout Clinical Subtype Questionnaire” (BCSQ-36/BCSQ-12)
Source: Int J Environ Res Public Health. 2020 Feb 8;17(3):1081. doi: 10.3390/ijerph17031081 (PMC7036968; doi:10.3390/ijerph17031081)
Supplement: Supplementary file 1 [file ijerph-17-01081-s001.zip › Table S1.docx]

**Table S1. Brazilian long BCSQ-36 and short BCSQ-12.**

As sentenças a seguir indicam uma série de experiências que podem ocorrer no ambiente de trabalho. Leia cada sentença cuidadosamente e marque com um X a opção que melhor representa o que você sente, o que você faz, e o que você pensa sobre o seu trabalho. Não existem respostas certas ou erradas. Por favor, **NÃO DEIXE NENHUMA SENTENÇA SEM RESPOSTA.**

**1** Totalmente em desacordo

**2** Muito em desacordo

**3** Em desacordo

**4** Indeciso

**5** De acordo

**6** Muito de acordo

**7** Totalmente de acordo

| 1. Eu tenho a necessidade de obter grandes triunfos no trabalho |  |
| --- | --- |
| 2. Eu acho que invisto mais do que o saudável em minha dedicação ao trabalho |  |
| 3. No trabalho, eu invisto todo o esforço necessário até que eu supere as dificuldades |  |
| 4. Eu tenho a ambição de obter grandes resultados no trabalho |  |
| 5. Eu descuido da minha vida pessoal ao perseguir grandes objetivos no trabalho |  |
| 6. Eu me envolvo com grande esforço na solução dos problemas de trabalho |  |
| 7. Eu sinto a necessidade de conseguir grandes metas no trabalho |  |
| 8. Eu arrisco minha saúde ao perseguir bons resultados no trabalho |  |
| 9. Se no trabalho eu não alcanço o resultado esperado, eu me empenho mais para alcançá-lo |  |
| 10. Eu tenho uma forte necessidade de grandes realizações no trabalho |  |
| 11. Eu ignoro minhas próprias necessidades para cumprir com as demandas do trabalho |  |
| 12. Diante de dificuldades no trabalho eu reajo com maior participação |  |
| 13. Eu me sinto indiferente e com pouca inclinação ao meu trabalho |  |
| 14. Eu gostaria de me dedicar a outro trabalho que apresentasse maiores desafios à minha capacidade |  |
| 15. Eu sinto que o meu trabalho é mecânico e rotineiro |  |
| 16. Eu tenho pouco interesse pelas tarefas do meu posto de trabalho |  |
| 17. Eu sinto que as minhas atividades laborais são um freio para o desenvolvimento de minhas capacidades |  |
| 18. O meu trabalho me oferece pouca variedade de atividades |  |
| 19. Eu não tenho ilusão pelo meu trabalho |  |
| 20. Eu gostaria de desempenhar outro trabalho no qual eu pudesse desenvolver melhor o meu talento |  |
| 21. Eu estou descontente no meu trabalho pela monotonia das tarefas |  |
| 22. No trabalho eu me comporto com despreocupação e desinteresse |  |
| 23. Meu trabalho não me oferece oportunidade para o desenvolvimento da minha aptidão |  |
| 24. Eu me sinto entediado no trabalho |  |
| 25. As pessoas que demandam meus serviços não demonstram apreço nem gratidão pelos meus esforços |  |
| 26. Quando as coisas do trabalho não saem tão bem quanto deveriam, eu deixo de me esforçar |  |
| 27. Eu me sinto impotente em muitas situações no meu trabalho |  |
| 28. O reconhecimento profissional independe do quanto uma pessoa se esforce no trabalho |  |
| 29. Quando encontro dificuldades no meu trabalho, eu me rendo |  |
| 30. Eu me sinto indefeso frente a algumas situações do meu trabalho |  |
| 31. Na organização onde eu trabalho não se leva em consideração o esforço e a dedicação |  |
| 32. Frente a qualquer dificuldade nas tarefas do meu trabalho, eu abandono |  |
| 33. Eu sinto que os resultados do meu trabalho escapam do meu controle |  |
| 34. Eu creio que a minha dedicação ao trabalho não é reconhecida |  |
| 35. Quando o esforço investido no trabalho não é suficiente, eu me dou por vencido |  |
| 36. No meu trabalho, eu trato com muitas situações que estão fora do meu controle |  |

**Correction algorithm:**  “Frenetic” scale is made up of 3 dimensions: ‘involvement’ (items nº 3, 6, 9, 12), ‘ambition’ (items nº 1, 4, 7, 10) and ‘**overload**’ (items nº 2, 5, 8, 11); “under-challenged” scale includes the following 3 dimensions: ‘indifference’ (items nº 13, 16, 19, 22), ‘**lack of development**’ (items nº 14, 17, 20, 23) and ‘boredom’ (items nº 15, 18, 21, 24); and worn-out scale is made up of 3 dimensions: ‘**neglect**’ (items nº 26, 29, 32, 35), ‘lack of acknowledgement (items nº 25, 28, 31, 34) and ‘lack of control’ (items nº 27, 30, 33, 36). The BCSQ-12 dimensions are in **bold**.
