# Supplementary material for: Frenetic, under-Challenged, and Worn-out Burnout Subtypes among Brazilian Primary Care Personnel: Validation of the Brazilian “Burnout Clinical Subtype Questionnaire” (BCSQ-36/BCSQ-12)
Source: Int J Environ Res Public Health. 2020 Feb 8;17(3):1081. doi: 10.3390/ijerph17031081 (PMC7036968; doi:10.3390/ijerph17031081)
Supplement: Supplementary file 1 [file ijerph-17-01081-s001.zip › Table S2.docx]

**Table S2. English long BCSQ-36 and short BCSQ-12.**

The following is a series of statements indicating experiences that may occur at work. Read each statement carefully and mark with an X the option that best represents how you feel, what you do and what you think about your work. There are no right or wrong answers. Please **DO NOT LEAVE ANY STATEMENT UNANSWERED.**

**1** Totally disagree

**2** Strongly disagree

**3** Disagree

**4** Unsure

**5** Agree

**6** Strongly agree

**7** Totally agree

| 1. I need to achieve great success in my work |  |
| --- | --- |
| 2. I think the dedication I invest in my work is more than what I should for my health |  |
| 3. I invest the necessary effort in my work until I overcome difficulties |  |
| 4. I am ambitious to obtain important results in my work |  |
| 5. I neglect my personal life when I pursue important achievements in my work |  |
| 6. I get very involved in solving work-related problems |  |
| 7. I feel the need to achieve important goals in my work |  |
| 8. I risk my health when I pursue good results in my work |  |
| 9. If I don’t achieve the expected result in my work, I try harder to achieve it |  |
| 10. I have a strong need for important achievements in my work |  |
| 11. I overlook my own needs to fulfil work demands |  |
| 12. I react to difficulties in my work with greater participation |  |
| 13. I feel indifferent about my work and have little desire to succeed |  |
| 14. I would like to be doing another job that is more challenging for my abilities |  |
| 15. I feel my work is mechanical and routine |  |
| 16. I have little interest for the tasks involved in my job |  |
| 17. I feel that my work is an obstacle to the development of my abilities |  |
| 18. My work offers me little variety in its activities |  |
| 19. I’m not enthusiastic about my work |  |
| 20. I would like to be doing another job where I can better develop my talents |  |
| 21. I am unhappy with my work because the tasks involved are monotonous |  |
| 22. I behave in a unconcerned and reluctant way at work |  |
| 23. My work doesn’t offer me opportunities to develop my abilities |  |
| 24. I feel bored at work |  |
| 25. The people who need my services don’t show appreciation or gratitude for my efforts |  |
| 26. When things at work don’t turn out as well as they should, I stop trying |  |
| 27. I feel helpless in many situations in my work |  |
| 28. Professional recognition doesn’t depend on efforts made at work |  |
| 29. I give up in response to difficulties in my work |  |
| 30. I feel defenseless in some situations in my work |  |
| 31. The organisation I work for doesn’t take notice of effort and dedication |  |
| 32. I give up in the face of any difficulties in my work tasks |  |
| 33. I feel the results of my work are beyond my control |  |
| 34. I think my dedication to my work is not acknowledged |  |
| 35. When the effort I invest in work is not enough, I give in |  |
| 36. I deal with many situations in my work that are beyond my control |  |

**Correction algorithm:**  “Frenetic” scale is made up of three dimensions: ‘involvement’ (items nº 3, 6, 9, 12), ‘ambition’ (items nº 1, 4, 7, 10) and ‘**overload’** (items nº 2, 5, 8, 11); “underchallenged” scale includes the following three dimensions: ‘indifference’ (items nº 13, 16, 19, 22), ‘**lack of development**’ (items nº 14, 17, 20, 23) and ‘boredom’ (items nº 15, 18, 21, 24); and wornout scale is made up of three dimensions: ‘**neglect’** (items nº 26, 29, 32, 35), ‘lack of acknowledgement (items nº 25, 28, 31, 34) and ‘lack of control’ (items nº 27, 30, 33, 36). The BCSQ-12 dimensions are in **bold**.
