# Supplementary material for: Frenetic, under-Challenged, and Worn-out Burnout Subtypes among Brazilian Primary Care Personnel: Validation of the Brazilian “Burnout Clinical Subtype Questionnaire” (BCSQ-36/BCSQ-12)
Source: Int J Environ Res Public Health. 2020 Feb 8;17(3):1081. doi: 10.3390/ijerph17031081 (PMC7036968; doi:10.3390/ijerph17031081)
Supplement: Supplementary file 1 [file ijerph-17-01081-s001.zip › Table S3.docx]

**Table S3. Socio-demographic and occupational factors related to the BCSQ-36 burnout subtypes in Brazilian primary care professionals.**

|  | **Frenetic** |  |  |  | **Under-challenged** |  |  |  | **Worn-out** |  |  |  |
| --- | --- | --- | --- | --- | --- | --- | --- | --- | --- | --- | --- | --- |
| **Factor** | **raw OR**  **(95% CI)** | ***p*** | **adj OR**  **(95% CI)** | ***p*** | **raw OR**  **(95% CI)** | ***p*** | **adj OR**  **(95% CI)** | ***p*** | **raw OR**  **(95% CI)** | ***p*** | **adj OR**  **(95% CI)** | ***p*** |
|  |  |  |  |  |  |  |  |  |  |  |  |  |
| **Age** |  |  |  |  |  |  |  |  |  |  |  |  |
|  |  |  |  |  |  |  |  |  |  |  |  |  |
| <35 years | ref. |  | - |  | ref. |  | - |  | ref. |  | ref. |  |
| 35-50 years | 1.25 (0.70-2.24) | .554 | - | - | 1.37 (0.76-2.46) | .300 | - | - | 1.61 (0.86-3.02) | .141 | 1.59 (0.80-3.18) | .187 |
| >50 years | 1.28 (0.68-2.40) | .452 | - | - | 1.48 (0.78-2.79) | .228 | - | - | 2.47 (1.28-4.77) | .007 | 4.58 (1.89-11.10) | **.001** |
|  |  |  |  |  |  |  |  |  |  |  |  |  |
| **Sex** |  |  |  |  |  |  |  |  |  |  |  |  |
| female | ref. |  | - |  | ref. |  | - |  | ref. |  | - |  |
| male | 0.67 (0.34-1.32) | .251 | - | - | 1.60 (0.90-2.85) | .113 | - | - | 1.26 (0.69-2.29) | .554 | - | - |
|  |  |  |  |  |  |  |  |  |  |  |  |  |
| **Relationship** |  |  |  |  |  |  |  |  |  |  |  |  |
| yes | ref. |  | - |  | ref. |  | - |  | ref. |  | - |  |
| no | 1.20 (0.74-1.95) | .456 | - | - | 1.47 (0.91-2.36) | .113 | - | - | 1.28 (0.79-2.07) | .319 | - | - |
|  |  |  |  |  |  |  |  |  |  |  |  |  |
| **Children** |  |  |  |  |  |  |  |  |  |  |  |  |
| none | ref. |  | - |  | ref. |  | - |  | ref. |  | - |  |
| one or more | 0.84 (0.53-1.32) | .439 | - | - | 0.92 (0.58-1.45) | .725 | - | - | 0.93 (0.59-1.47) | .761 | - | - |
|  |  |  |  |  |  |  |  |  |  |  |  |  |
| **Category** |  |  |  |  |  |  |  |  |  |  |  |  |
| volunteer | ref. |  | ref. |  | ref. |  | ref. |  | ref. |  | ref. |  |
| professional | 1.70 (1.05-2.76) | .031 | 1.68 (1.03-2.76) | **.040** | 1.70 (1.05-2.76) | .031 | 1.70 (1.04-2.77) | **.034** | 1.60 (0.99-2.59) | .054 | 1.20 (0.69-2.10) | .514 |
|  |  |  |  |  |  |  |  |  |  |  |  |  |
| **Job position** |  |  |  |  |  |  |  |  |  |  |  |  |
| psysician | ref. |  | - |  | ref. |  | - |  | ref. |  | ref. |  |
| nurse | 0.80 (0.40-1.59) | .526 | - | - | 0.99 (0.47-2.07) | .969 | - | - | 0.36 (0.18-0.72) | .004 | 0.35 (0.15-0.79) | **.011** |
| CHV | 0.84 (0.46-1.53) | .572 | - | - | 1.53 (0.81-2.90) | .187 | - | - | 0.47 (0.27-0.83) | .009 | 0.51 (0.25-1.05) | .068 |
|  |  |  |  |  |  |  |  |  |  |  |  |  |
| **Hours per week** |  |  |  |  |  |  |  |  |  |  |  |  |
| <40 hours | ref. |  | ref. |  | ref. |  | - |  | ref. |  | ref. |  |
| 40 hours | 1.67 (0.85-3.29) | .140 | 1.78 (0.90-3.52) | .100 | 0.94 (0.41-2.14) | .882 | - | - | 0.46 (0.21-1.00) | .051 | 1.48 (0.74-2.97) | .271 |
| >40 hours | 2.44 (1.09-4.49) | .031 | 2.31 (1.02-5.21) | **.044** | 1.34 (0.70-2.56) | .382 | - | - | 0.60 (0.33-1.08) | .087 | 1.94 (0.83-4.56) | .129 |
|  |  |  |  |  |  |  |  |  |  |  |  |  |
| **Length of service** |  |  |  |  |  |  |  |  |  |  |  |  |
| <6 years | ref. |  | - |  | ref. |  | - |  | ref. |  | ref. |  |
| 6-16 years | 1.02 (0.49-2.10) | .962 | - | - | 1.17 (0.57-2.40) | .667 | - | - | 2.46 (1.12-5.42) | .025 | 6.11 (2.37-15.78) | **<.001** |
| >16 years | 1.19 (0.59-2.38) | .631 | - | - | 1.13 (0.56-2.26) | .741 | - | - | 1.46 (0.66-3.21) | .349 | 7.07 (2.32-21.59) | **.001** |
|  |  |  |  |  |  |  |  |  |  |  |  |  |
| **Length same job** |  |  |  |  |  |  |  |  |  |  |  |  |
| <6 years | ref. |  | - |  | ref. |  | ref. |  | ref. |  | - |  |
| 6-16 years | 0.91 (0.55-1.50) | .717 | - | - | 1.23 (0.75-2.00) | .415 | 0.98 (0.59-1.63) | .941 | 1.04 (0.63-1.70) | .886 | - | - |
| >16 years | 1.14 (0.39-3.31) | .814 | - | - | 2.68 (1.01-7.08) | .047 | 1.13 (0.39-3.30) | .826 | 1.18 (0.41-3.45) | .757 | - | - |
|  |  |  |  |  |  |  |  |  |  |  |  |  |
| **Contract period** |  |  |  |  |  |  |  |  |  |  |  |  |
| temporary | ref. |  | - |  | ref. |  | - |  | ref. |  | - |  |
| permanent | 0.72 (0.35-1.48) | .367 | - | - | 1.35 (0.60-3.04) | .470 | - | - | 0.72 (0.35-1.48) | .367 | - | - |
|  |  |  |  |  |  |  |  |  |  |  |  |  |
| **Contract type** |  |  |  |  |  |  |  |  |  |  |  |  |
| full-time | ref. |  | - |  | ref. |  | - |  | ref. |  | - |  |
| part-time | 0.84 (0.39-1.82) | .655 | - | - | 1.09 (0.53-2.27) | .813 | - | - | 1.29 (0.63-2.63) | .487 | - | - |
|  |  |  |  |  |  |  |  |  |  |  |  |  |
| **Economic dificulties** |  |  |  |  |  |  |  |  |  |  |  |  |
| never | ref. |  | - |  | ref. |  | - |  | ref. |  | - |  |
| sometimes | 1.37 (0.67-2.78) | .385 | - | - | 0.63 (0.31-1.27) | .194 | - | - | 0.61 (0.30-1.21) | .155 | - | - |
| almost always | 1.27 (0.60-2.68) | .529 | - | - | 1.39 (0.70-2.78) | .348 | - | - | 1.34 (0.68-2.64) | .402 | - | - |
| always | 1.60 (0.73-3.50) | .240 | - | - | 1.56 (0.75-3.26) | .235 | - | - | 1.06 (0.50-2.23) | .881 | - | - |
|  |  |  |  |  |  |  |  |  |  |  |  |  |
| **Sick Leave** |  |  |  |  |  |  |  |  |  |  |  |  |
| yes | ref. |  | - |  | ref. |  | ref. |  | ref. |  | ref. |  |
| no | 1.06 (0.66-1.70) | .814 | - | - | 0.63 (0.40-0.99) | .049 | 1.12 (0.69-1.80) | .646 | 0.37 (0.23-0.58) | <.001 | 0.39 (0.24-0.64) | **<.001** |
|  |  |  |  |  |  |  |  |  |  |  |  |  |

Raw OR: Odds Ratio resulting from bivariate logistic regression models. Adj-OR: Adjusted OR from multivariate logistic regression. 95% CI: 95% confidence interval. Ref.= reference category. CHV: community health workers
