# Supplementary material for: Frenetic, under-Challenged, and Worn-out Burnout Subtypes among Brazilian Primary Care Personnel: Validation of the Brazilian “Burnout Clinical Subtype Questionnaire” (BCSQ-36/BCSQ-12)
Source: Int J Environ Res Public Health. 2020 Feb 8;17(3):1081. doi: 10.3390/ijerph17031081 (PMC7036968; doi:10.3390/ijerph17031081)
Supplement: Supplementary file 1 [file ijerph-17-01081-s001.zip › Table S4.docx]

**Table S4. Socio-demographic and occupational factors related to the BCSQ-12 subscales in Brazilian primary care professionals.**

|  | **Overload** |  |  |  | **L. Development** |  |  |  | **Neglect** |  |  |  |
| --- | --- | --- | --- | --- | --- | --- | --- | --- | --- | --- | --- | --- |
| **Factor** | **raw OR**  **(95% CI)** | ***p*** | **adj OR**  **(95% CI)** | ***p*** | **raw OR**  **(95% CI)** | ***p*** | **adj OR**  **(95% CI)** | ***p*** | **raw OR**  **(95% CI)** | ***p*** | **adj OR**  **(95% CI)** | ***p*** |
|  |  |  |  |  |  |  |  |  |  |  |  |  |
| **Age** |  |  |  |  |  |  |  |  |  |  |  |  |
|  |  |  |  |  |  |  |  |  |  |  |  |  |
| <35 years | ref. |  | - |  | ref. |  | ref. |  | ref. |  | ref. |  |
| 35-50 years | 1.05 (0.60-1.85) | .866 | - | - | 1.27 (0.69-2.31) | .444 | 1.17 (0.63-2.18) | .624 | 1.12 (0.62-2.03) | .713 | 0.97 (0.50-1.86) | .922 |
| >50 years | 0.98 (0.53-1.83) | .950 | - | - | 1.85 (0.98-3.49) | .057 | 2.10 (1.08-4.09) | .**030** | 1.81 (0.97-3.37) | **.064** | 1.56 (0.69-3.54) | .288 |
|  |  |  |  |  |  |  |  |  |  |  |  |  |
| **Sex** |  |  |  |  |  |  |  |  |  |  |  |  |
| female | ref. |  | - |  | ref. |  | ref. |  | ref. |  | - |  |
| male | 0.59 (0.29-1.18) | .133 | - | - | 1.65 (0.92-2.95) | .091 | 2.20 (1.14-4.25) | **.019** | 1.26 (0.69-2.29) | .454 | - | - |
|  |  |  |  |  |  |  |  |  |  |  |  |  |
| **Relationship** |  |  |  |  |  |  |  |  |  |  |  |  |
| yes | ref. |  | - |  | ref. |  | - |  | ref. |  | ref. |  |
| no | 0.86 (0.52-1.42) | .196 | - | - | 1.36 (0.84-2.19) | .213 | - | - | 1.82 (1.14-2.92) | **.013** | 1.71 (1.05-2.78) | **.032** |
|  |  |  |  |  |  |  |  |  |  |  |  |  |
| **Children** |  |  |  |  |  |  |  |  |  |  |  |  |
| none | ref. |  | - |  | ref. |  | - |  | ref. |  | - |  |
| one or more | 0.95 (0.60-1.51) | .833 | - | - | 0.80 (0.48-1.18) | .214 | - | - | 0.79 (0.50-1.25) | .313 | - | - |
|  |  |  |  |  |  |  |  |  |  |  |  |  |
| **Category** |  |  |  |  |  |  |  |  |  |  |  |  |
| volunteer | ref. |  | ref. |  | ref. |  | - |  | ref. |  | - |  |
| professional | 1.54 (0.96-2.48) | .076 | 1.51 (0.92-2.49) | .104 | 0.96 (0.60-1.52) | .852 | - | - | 0.69 (0.44-1.09) | .111 | - | - |
|  |  |  |  |  |  |  |  |  |  |  |  |  |
| **Job position** |  |  |  |  |  |  |  |  |  |  |  |  |
| psysician | ref. |  | - |  | ref. |  | ref. |  | ref. |  | - |  |
| nurse | 0.90 (0.47-1.75) | .762 | - | - | 1.62 (0.75-3.49) | .215 | 2.08 (0.85-5.06) | .107 | 0.87 (0.43-1.77) | .708 | - | - |
| CHV | 0.64 (0.35-1.15) | .134 | - | - | 1.89 (0.96-3.75) | .067 | 2.10 (0.94-4.69) | .069 | 1.04 (0.57-1.91) | .898 | - | - |
|  |  |  |  |  |  |  |  |  |  |  |  |  |
| **Hours per week** |  |  |  |  |  |  |  |  |  |  |  |  |
| <40 hours | ref. |  | ref. |  | ref. |  | ref. |  | ref. |  | - |  |
| 40 hours | 1.30 (0.68-2.48) | .428 | 1.43 (0.74-2.78) | .287 | 1.87 (0.95-3.68) | .069 | 1.83 (0.91-3.66) | .090 | 1.10 (0.59-2.05) | .768 | - | - |
| >40 hours | 2.32 (1.07-5.05) | .033 | 2.69 (1.20-6.06) | **.017** | 1.53 (0.66-3.57) | .325 | 1.56 (0.64-3.82) | .332 | 1.48 (0.68-3.20) | .322 | - | - |
|  |  |  |  |  |  |  |  |  |  |  |  |  |
| **Length of service** |  |  |  |  |  |  |  |  |  |  |  |  |
| <6 years | ref. |  | ref. |  | ref. |  | - |  | ref. |  | ref. |  |
| 6-16 years | 1.73 (0.77-3.85) | .181 | 1.72 (0.74-4.01) | .207 | 1.09 (0.63-1.89) | .750 | - | - | 0.82 (0.48-1.40) | .472 | 1.80 (0.83-3.92) | .138 |
| >16 years | 2.02 (0.93-4.40) | .076 | 2.14 (0.91-5.05) | **.**083 | 0.91 (0.51-1.62) | .757 | - | - | 0.57 (0.31-1.01) | **.056** | 1.05 (0.42-2.63) | .911 |
|  |  |  |  |  |  |  |  |  |  |  |  |  |
| **Length same job** |  |  |  |  |  |  |  |  |  |  |  |  |
| <6 years | ref. |  | ref. |  | ref. |  | - |  | ref. |  | - |  |
| 6-16 years | 1.50 (0.93-2.43) | .098 | 1.35 (0.80-2.27) | .266 | 1.01 (0.62-1.67) | .959 | - | - | 0.89 (0.54-1.47) | .647 | - | - |
| >16 years | 1.75 (0.63-4.85) | .285 | 1.55 (0.53-4.52) | .425 | 2.00 (0.74-5.37) | .170 | - | - | 1.92 (0.72-5.15) | .196 | - | - |
|  |  |  |  |  |  |  |  |  |  |  |  |  |
| **Contract period** |  |  |  |  |  |  |  |  |  |  |  |  |
| temporary | ref. |  | - |  | ref. |  | - |  | ref. |  | - |  |
| permanent | 0.64 (0.32-1.29) | .213 | - | - | 1.11 (0.51-2.43) | .792 | - | - | 1.11 (0.51-2.43) | .792 | - | - |
|  |  |  |  |  |  |  |  |  |  |  |  |  |
| **Contract type** |  |  |  |  |  |  |  |  |  |  |  |  |
| full-time | ref. |  | - |  | ref. |  | - |  | ref. |  | - |  |
| part-time | 0.96 (0.45-2.04) | .917 | - | - | 0.60 (0.26-1.39) | .230 | - | - | 1.12 (0.54-2.34) | .753 | - | - |
|  |  |  |  |  |  |  |  |  |  |  |  |  |
| **Economic dificulties** |  |  |  |  |  |  |  |  |  |  |  |  |
| never | ref. |  | - |  | ref. |  | ref. |  | ref. |  | ref. |  |
| sometimes | 1.23 (0.60-2.51) | .568 | - | - | 0.90 (0.43-1.87) | .769 | 0.83 (0.39-1.78) | .631 | 1.51 (0.70-3.29) | .296 | 1.53 (0.69-3.38) | .295 |
| almost always | 1.60 (0.77-3.33) | .209 | - | - | 1.74 (0.84-3.62) | .135 | 1.80 (0.82-3.93) | .141 | 2.30 (1.05-5.05) | .**038** | 2.53 (1.12-5.72) | .**025** |
| always | 1.50 (0.68-3.30) | .313 | - | - | 2.04 (0.94-4.40) | .070 | 1.97 (0.87-4.48) | .106 | 2.64 (1.16-6.04) | **.021** | 2.70 (1.16-6.29) | **.021** |
|  |  |  |  |  |  |  |  |  |  |  |  |  |
| **Sick Leave** |  |  |  |  |  |  |  |  |  |  |  |  |
| yes | ref. |  | - |  | ref. |  | - |  | ref. |  | - |  |
| no | 0.73 (0.46-1.15) | .177 | - | - | 0.75 (0.48-1.20) | .230 | - | - | 0.80 (0.50-1.27) | .336 | - | - |
|  |  |  |  |  |  |  |  |  |  |  |  |  |

Raw OR: Odds Ratio resulting from bivariate logistic regression models. Adj-OR: Adjusted OR from multivariate logistic regression. 95% CI: 95% confidence interval. Ref.= reference category. CHV: community health workers.
